# Line-field confocal optical coherence tomography assessment of pityriasis rosea

**DOI:** 10.1016/j.jdcr.2023.06.049

**Published:** 2023-07-19

**Authors:** Gaurav N. Pathak, Thu M. Truong, Babar K. Rao

**Affiliations:** a Rao Dermatology, Atlantic Highlands, New Jersey; b Department of Dermatology, Rutgers Robert Wood Johnson Medical School, Somerset, New Jersey; c Department of Dermatology, Weill Cornell Medicine, New York, New York

**Keywords:** confocal imaging, LC-OCT, line-field confocal optical coherence tomography, pityriasis rosea, spongiotic dermatitis

## Clinical presentation

A healthy 24-year-old man presented with a discrete erythematous scaly plaque on the right mid-back associated with pruritus for 4 weeks. On physical examination, in addition to the plaque, there were several smaller papules scattered on the right mid-back and left mid-back. His family medical history was significant for pemphigus vulgaris and myasthenia gravis. Based on his clinical presentation and associated signs/symptoms, there was a clinical suspicion of psoriasis. A 2-cm punch biopsy was performed.

## Clinical appearance

**Fig 1:**
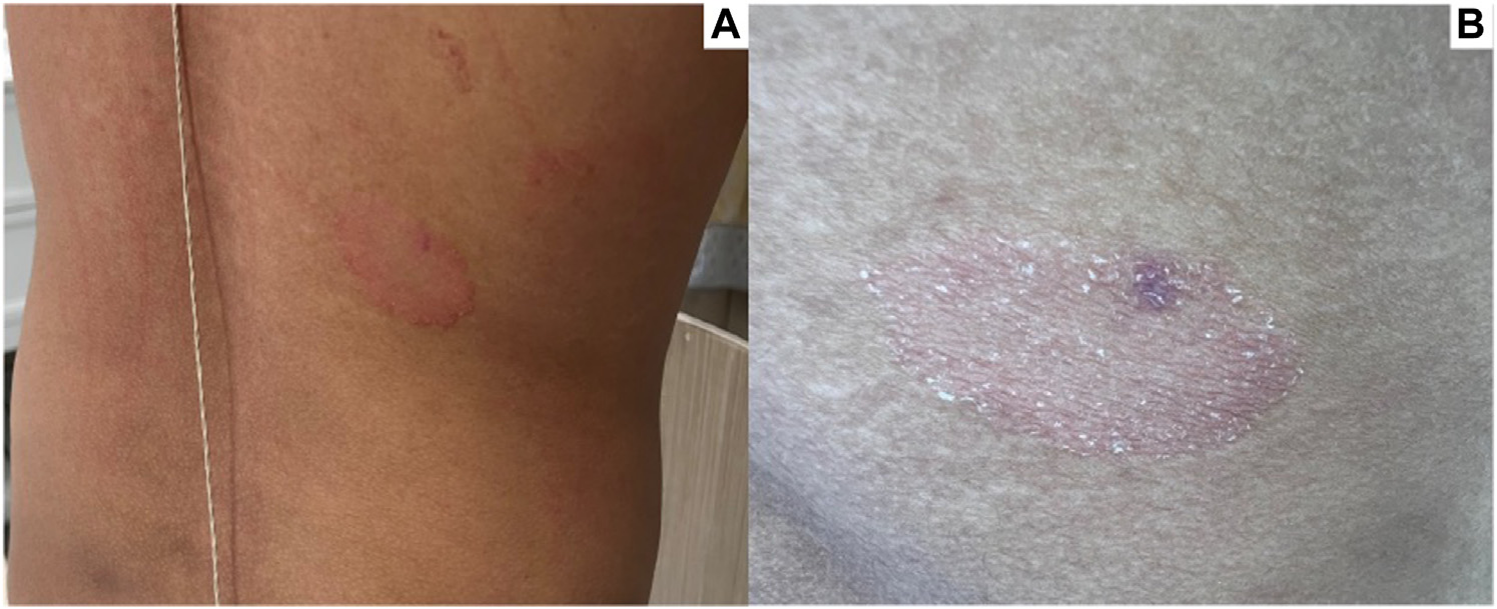
**A,** A 2.5-cm red plaque on the mid-right back region. **B,** Plaque demonstrating a raised border and a depressed center with fine scale. The hyperpigmented macule was the site of punch biopsy.

A well-defined, annular, scaly, pink plaque with a raised border and a depressed center (Fig 1) is located on the right mid-back.

## Line-field confocal optical coherence tomography appearance

**Fig 2:**
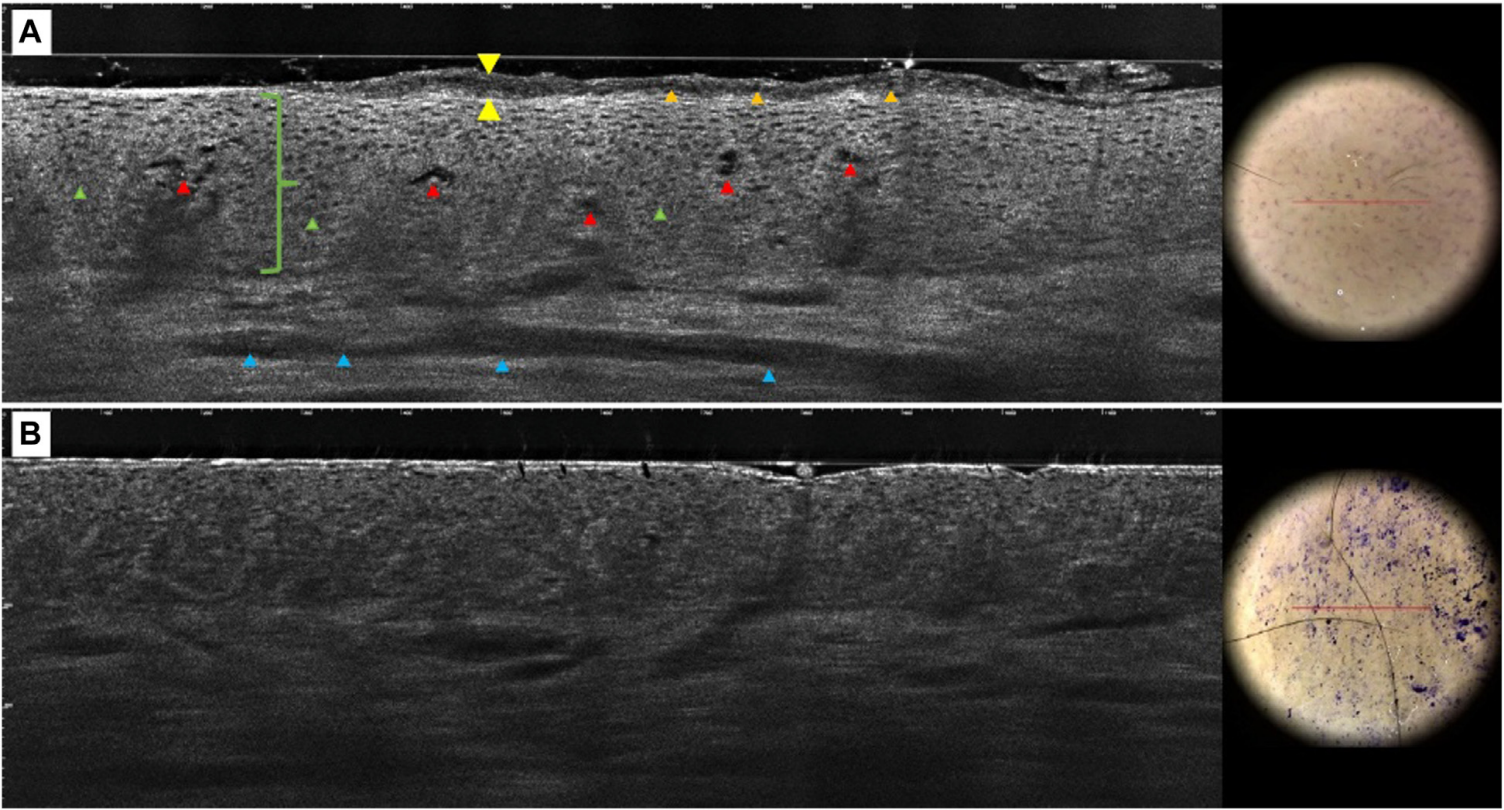
Two-dimensional vertical view of the mid-back. **A,** Digital histology image of a plaque with hyperkeratosis (*yellow arrows*), patchy parakeratosis (*orange arrows*), acanthosis (*green bracket*), spongiosis (*green arrows*), dilated blood vessels (*blue arrows*), and possible extravasation of red blood cells, also called “hemorrhage” (*red arrows*). **B,** Digital histology image of adjacent area with no visible lesion lacking features defined in image **(A)**. The presence of patchy parakeratosis and hemorrhage is suggestive of pityriasis rosea.

**Fig 3:**
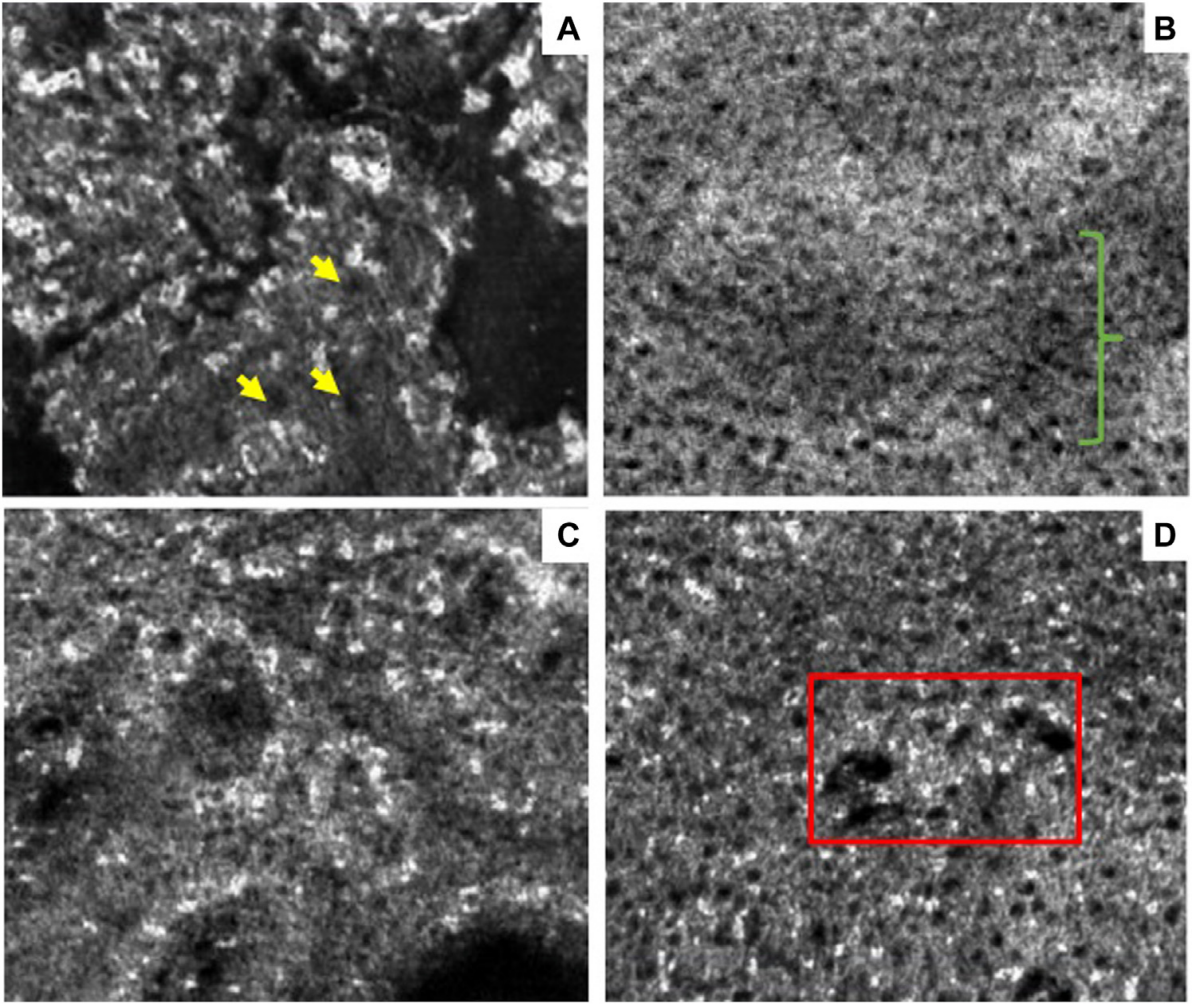
Line-field confocal optical coherence tomography (LC-OCT) (en face) view. **A,** Hyperkeratosis with focal parakeratosis (*yellow arrows*) in stratum corneum at 5-μm depth. **B,** Darker epidermal areas (*green bracket*) with presence of a few small bright inflammatory cells within the epidermis at 35-μm depth. **C,** Dermoepidermal junction at 75-μm depth. **D,** Tips of the dermal papillae with dark globular aggregates likely representing erythrocytes (*red box*) at 117-μm depth.

A confocal microscopy assessment was obtained using line-field confocal optical coherence tomography (LC-OCT). In the two-dimensional vertical (histologic) view (Fig 2) and “en face” views (Fig 3), hyperkeratosis was seen in the form of thickened bright structures in addition to dark polygonal structures corresponding to parakeratosis. Acanthosis was seen as islands of keratinocytes with broadened outlines and dark rounded areas corresponding to spongiosis. There were focal areas of dark globular structures at the tips of the dermal papillae, possibly corresponding to erythrocyte extravasates or dilated capillaries with infiltrate.

## Histologic diagnosis

**Fig 4:**
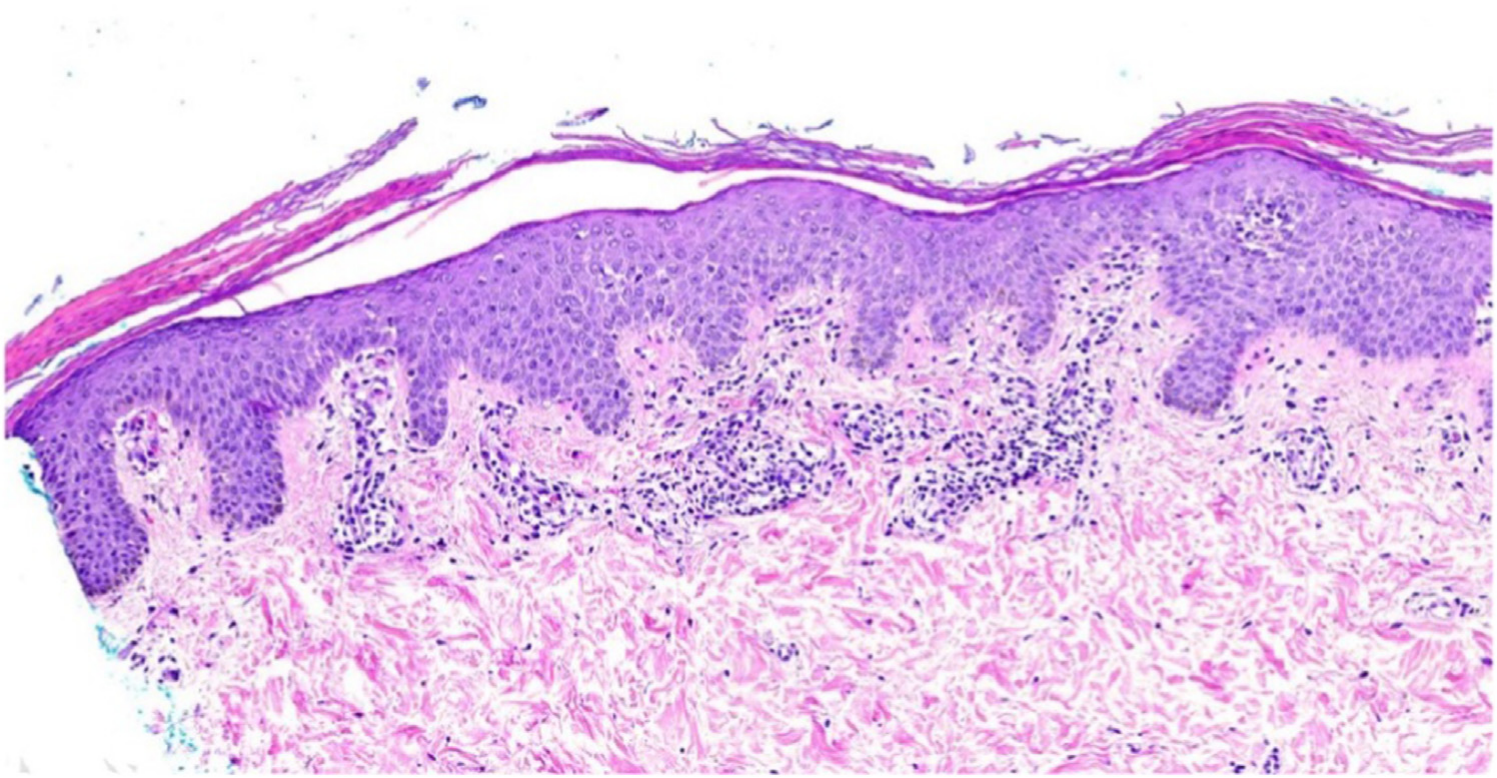
Punch biopsy of the scaly plaque.

The punch biopsy showed a subacute spongiotic process (Fig 4). Epidermal hyperkeratosis, acanthosis, and mild spongiosis were present. There were foci of parakeratosis with mild infiltration of mononuclear cells and extravasation of erythrocytes within the papillary dermis. These features of spongiotic dermatitis are consistent with pityriasis rosea (PR) based on the clinical presentation.

## Key message

LC-OCT is a noninvasive, in vivo, high-resolution imaging technique that provides information regarding the epidermal and superficial- to mid-dermal architecture. It captures vertical and horizontal two-dimensional images to create a three-dimensional image block. It has been used to evaluate histopathologic features of skin cancer, pustular dermatoses, and some eczematous conditions.^1^

Although PR may clinically mimic other eczematous dermatoses, which present as erythematous papules and plaques, rapid histopathologic visualization with LC-OCT can help determine the diagnosis. Key differentials that appear histologically as spongiotic dermatitis include psoriasis, contact dermatitis, and nummular eczema. A lack of visualization by LC-OCT of psoriasiform dermatitis, papillomatosis, and Munro microabscesses (usually visible in the epidermis as dark roundish areas with bright particles corresponding to neutrophils) makes the diagnosis of psoriasis less likely. In acute spongiotic processes, like allergic or irritant contact dermatitis, the widening of intercellular spaces between the keratinocytes forms epidermal vesicles.^2^ Parakeratosis is usually present, and lymphocytic/leukocytic infiltration can also occur. The absence of vesicles makes an acute spongiotic process less likely. While spongiosis and parakeratosis are present in nummular eczema, the presence of focal parakeratosis with hemorrhage supports the diagnosis of PR.

In summary, we found good histopathologic correlation of LC-OCT visualization with key findings of focal parakeratosis, spongiosis, and erythrocyte extravasation in both “en face” and vertical views. This case report supports the utility of LC-OCT imaging as a clinical diagnostic aid for diagnosing PR. Future studies should evaluate LC-OCT imaging in other spongiotic dermatoses and in larger cohorts of patients with PR.

## Conflicts of interest

Dr Rao is a speaker for Incyte. All other authors have no disclosures.

## References

[1] Tognetti L., Cinotti E., Falcinelli F. (2022). Line-field confocal optical coherence tomography: a new tool for non-invasive differential diagnosis of pustular skin disorders. J Eur Acad Dermatol Venereol.

[2] Leung A.K.C., Lam J.M., Leong K.F., Hon K.L. (2021). Pityriasis rosea: an updated review. Curr Pediatr Rev.

